# Survival Disparities Between Patients with Breast Cancer With and Without HIV: A Research Framework

**DOI:** 10.1200/GO.22.00330

**Published:** 2023-04-19

**Authors:** Steady Chasimpha, Isabel dos Santos Silva, Yehoda M. Martei, Surbhi Grover, Herbert Cubasch, Valerie McCormack

**Affiliations:** ^1^ Department of Non-Communicable Disease Epidemiology, London School of Hygiene and Tropical Medicine, London, United Kingdom; ^2^ Department of Medicine (Hematology-Oncology), University of Pennsylvania, Philadelphia, PA; ^3^ Department of Radiation Oncology, University of Pennsylvania, Philadelphia, PA; ^4^ Department of Surgery, University of Witwatersrand, Johannesburg, South Africa; ^5^ Chris Hani Baragwanath Academic Hospital, Johannesburg, South Africa; ^6^ Environment and Lifestyle Epidemiology Branch, International Agency for Research on Cancer, Lyon, France

## Introduction

Because of potent antiretroviral therapy (ART), most women infected with HIV now live longer to ages when breast cancer incidence rates are high.^1^ Although not at increased risk of breast cancer, patients with HIV and breast cancer experience lower survival compared with their HIV-uninfected counterparts. A large US cancer- and HIV-linked registry-based study of patients with cancer, the HIV/AIDS-Cancer Match study, showed that all-cause mortality and breast cancer–specific mortality were 4.6 times (hazard ratio [HR]; 95% CI, 3.9 to 5.5) and 2.6 times (2.1-3.3), respectively, higher among patients with HIV and breast cancer (n = 314) than among patients with breast cancer uninfected by HIV, after adjusting for ethnicity and age, year of diagnosis, and tumor stage at cancer diagnosis.^2^ Similarly, in Sub-Saharan Africa (SSA) where breast cancer survival is on average lower than that in high-income countries,^3-5^ we found that patients with breast cancer and HIV experience lower overall survival than patients with breast cancer uninfected by HIV. The absolute 3-year overall survival was 9% lower for patients with HIV and breast cancer (HR, 46%; 95% CI, 40 to 53) versus (HR, 55%; 95% CI, 52 to 59) for patients with breast cancer uninfected by HIV in the African Breast Cancer-Disparities in Outcomes (ABC-DO) study.^5,6^ The ABC-DO study and South African Breast Cancer and HIV Outcomes (SABCHO) study,^7^ to our knowledge, the two largest prospective cohorts of women newly diagnosed with breast cancer in SSA (n = 313 and 600 patients with HIV, respectively, with a small number contributing to both cohorts) reported age- and stage-adjusted all-cause mortality HRs in HIV-infected versus HIV-uninfected patients with breast cancer of 1.41 (95% CI, 1.15 to 1.74)^6^ and 1.50 (95% CI, 1.22 to 1.85),^8^ respectively.

The direct/indirect impacts of the convergence of HIV and breast cancer on the management and outcomes of these two diseases are under-researched, and the underlying mechanisms for these cancer survival disparities by HIV status remain unclear. Existing research has been limited to either studies too small and underpowered to examine contributory factors or to studies that are large but lack the granularity needed to properly investigate these survival determinants.

We propose a research framework to highlight epidemiologic considerations that researchers might consider when investigating the extent of, and reasons for, breast cancer survival disparities by HIV status, especially in SSA where most patients with HIV reside. The framework may be modified to reflect context-specific issues in other settings. We hope that it will provide a foundation for harmonized research to address critical knowledge gaps in HIV-associated survival disparities among patients with breast cancer.

## Mortality Outcomes

**FIG 1:**
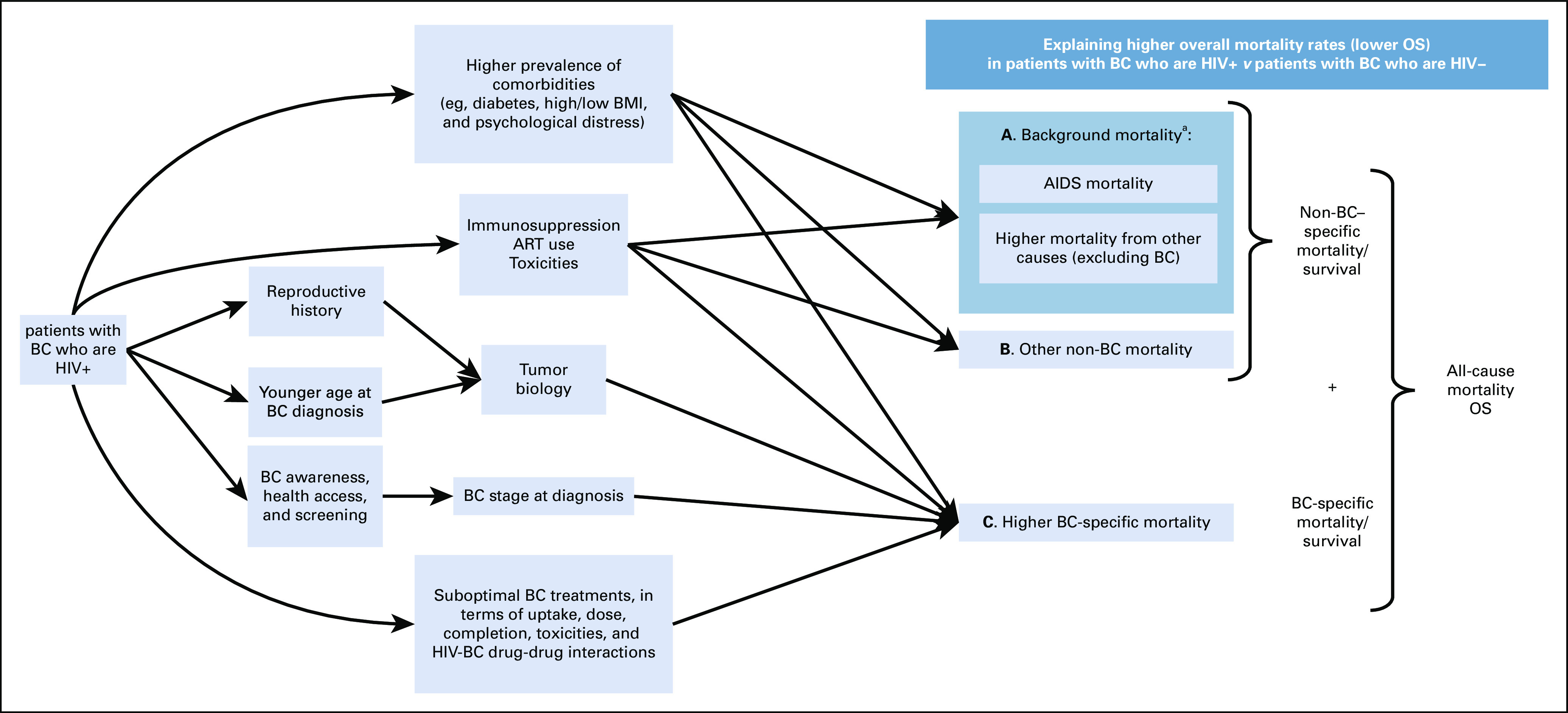
Potential pathways leading to higher overall mortality rates (lower OS) in patients with HIV and breast cancer. ^a^Taken into account through net survival. ART, antiretroviral therapy; BC, breast cancer; OS, overall survival.

Higher all-cause mortality rates in HIV-infected versus HIV-uninfected patients with breast cancer can be apportioned to different causes of deaths (Fig 1). First, patients with HIV and breast cancer might have higher rates of breast cancer–specific mortality than patients with breast cancer uninfected by HIV (Box C). Second, patients with HIV and breast cancer might have higher breast cancer–unrelated mortality than their HIV-uninfected counterparts (Box A + B), that is, because of deaths from AIDS and other non–breast cancer causes. These deaths include the higher background mortality rate of HIV-infected women than that of the female general population (Box A) and, possibly, rates of excess background mortality (Box B). Thus, the outcome definition is critical here and will affect the interpretation of findings, be it all-cause mortality, breast cancer–specific mortality, or other-cause mortalities. In the United States, analyzing all-cause mortality, Coghill et al^9^ found excess mortality rates among patients with HIV and breast cancer who were non-White and under age 70 years, that is, an excess above that of the combined effects of background mortality, mortality associated with HIV and with having breast cancer. Because drivers of mortality differences include not only biologic but also setting-specific factors, similar work in other settings is warranted.

Higher breast cancer–specific mortality in HIV-infected versus HIV-uninfected patients with breast cancer (Box C) may occur for multiple reasons, including if patients with HIV (1) have less favorable breast tumor characteristics at diagnosis or (2) have poorer access or adherence to standard cancer-directed treatment or suffer intensified adverse effects and HIV-cancer drug-to-drug interactions (these pathways are expanded on below). Indeed, findings from US studies with information on cause of death reported that patients with HIV and breast cancer had higher breast cancer–specific mortality compared with their HIV-uninfected counterparts (adjusted HR ranging from 1.85 to 2.84^2,10,11^). Equivalent data are not available from SSA because of low-quality data on the cause of death.

The lower overall survival in HIV-infected versus HIV-uninfected patients with breast cancer may be partially because patients with HIV have higher background mortality than their HIV-uninfected counterparts in the general population, because of AIDS deaths or higher non-AIDS mortality (eg, because of higher prevalence of comorbidities such as diabetes or high/low BMI; Box A). Even in this era of ART, life expectancy in patients with HIV (with and without breast cancer) remains lower than that of the general population.^12-15^ In addition, the copresence of HIV and breast cancer may lead to even higher non–breast cancer mortality (eg, because of psychological distress, suicide, and challenges of adherence to HIV treatment amid cancer therapy) than in the population of women with HIV only (Box B).

Differences in overall survival between HIV-infected versus HIV-uninfected patients with breast cancer use the all-cause mortality outcome (Box A + B + C to give HR_overall_). If net/relative survival methods are used, they examine all-cause mortality, adjusted for background mortality, by accounting for deaths that would have occurred over the follow-up period in a similar age- and HIV-specific population of women without breast cancer (ie, removing differences because of Box A, giving HR_net_). These net/relative survival methods use lifetables as the source of background mortality. Construction of lifetables requires information on observed age- and sex-specific mortality rates from national civil registration and vital statistics (CRVS) systems. In SSA, CRVS are often inadequate (often concentrated on cities, recorded cause of death information may be inaccurate and registered deaths are often not medically certified^15,16^). As such, mortality statistics/lifetables for most countries in SSA are calculated largely using prediction models and estimation procedures. In addition, estimating relative survival in the present context requires HIV-specific lifetables. The United Nations Joint Programme on HIV/AIDS produces annually updated mortality rates for the HIV-infected population, which can be used to generate national HIV-specific lifetables. However, these are also modeled mortality estimates,^16^ predominantly on the basis of data from surveillance of pregnant women (15-49 years old) attending sentinel antenatal clinics or Prevention of Mother to Child Transmission programs. Thus, they will be most reliable in younger age groups (<35 years), whereas in SSA, the current peak age at breast cancer diagnosis for patients infected with HIV ranges from 35 to 49 years.^17^ HIV-associated differentials will be larger in overall survival than in net survival (ie, HR_overall_ > HR_net_). Among HIV-infected and HIV-uninfected women separately, differences in overall and net survival will greatly depend on mean survival after breast cancer. Higher background mortality may be sizable relative to few deaths from breast cancer in a setting of high mean survival, but may be negligible in a setting of low mean survival (regardless of HIV status), as is typically the case in SSA.

## Breast Tumor Characteristics

Age, tumor stage, and tumor molecular subtype at breast cancer diagnosis may contribute to breast cancer–specific mortality differences if they differ substantially by HIV status. Currently, patients with HIV are diagnosed with breast cancer at younger ages compared with patients uninfected with HIV (approximately 10 years younger)^18,19^ because patients with HIV are younger than HIV-uninfected women in the general population. However, it remains unclear whether HIV predisposes patients with HIV to early onset of breast cancer, an issue that may be setting-specific, and although initial findings do not suggest a difference,^6,20-22^ further data on whether breast cancer subtypes differ by HIV are needed. Nevertheless, it is well established that younger patients with breast cancer uninfected by HIV present with more aggressive tumor subtypes, leading to poor prognosis and high recurrence rates.^23,24^ Previous studies in SSA have shown no differences in tumor molecular subtypes by HIV status,^6,21,22,25,26^ but this evidence is insufficient as there is limited routine access to high-quality immunohistochemistry services in most SSA.

Disparities in tumor stage at diagnosis by HIV status may vary between settings if they primarily reflect differences in breast cancer awareness, screening uptake, and/or disparities in health access or other social determinants of health. In the United States, patients with HIV might have lower access to screening or may develop breast cancer at younger ages than those targeted by screening or might have a higher incidence of interval cancers.^27^ Screening for breast cancer, either opportunistic or population-based, is rather limited in SSA. Studies in the United States suggest that patients with HIV are twice as likely to present with advanced-stage (TNM stage IV) breast cancer as women without HIV,^27,28^ but no such differences have been observed in SSA.^29^

## HIV-Cancer Treatment and Toxicities

Pathways leading to higher breast cancer–specific mortality in patients with HIV may additionally reflect potential differences in treatment by HIV status (Fig 1). Adequacy of multimodality cancer treatment, including relative dose intensity for systemic treatment, has been associated with survival outcomes in patients with breast cancer, especially those with early-stage disease treated with curative intent. Breast cancer treatment completion may be an important mediator of the association between HIV and breast cancer survival outcomes, but treatment data are often critically missing or only available as a dichotomous variable (eg, treated *v* untreated) in studies evaluating these associations.^11,21,22,30^ Key variables in standardized data collection tools should include complete information on systemic treatment including granular details on doses administered and dates of chemotherapy, timing and adequacy of curative surgery and total dose, timing and frequency of radiotherapy, type of ART, and virologic outcomes. These data will help investigate the contributory effects of treatment and toxicity and are actionable for reducing disparities in mortality/survival outcomes that may arise because of HIV-related differences in quality-of-cancer care being delivered, its uptake, and adherence.

Patients with HIV and breast cancer may also experience adverse effects of ART and cancer drug-to-drug interactions (DDIs), and lack of access to granulocyte colony-stimulating factors might lead to intensified treatment-related toxicities and side effects compared with their HIV-uninfected counterparts. ART regimens/agents, such as zidovudine (AZT—which is associated with high incidence of side effects such as anemia and neutropenia^31,32^), may influence chemotherapy by enhancing toxicity.^33^ In addition, chemotherapeutic agents and HIV drugs might have overlapping toxicities because of DDIs, affecting dosage in patients with HIV.^34,35^ In the AIDS Malignancy Consortium, patients with solid or hematologic tumors treated with sunitinib and on ritonavir protease inhibitor (PI)–based therapy experienced increased toxicity, including higher rates of grade 3 neutropenia, diarrhea, mucositis, and fatigue, even with reduced doses of sunitinib than patients on non–ritonavir PI-based therapy.^36^ There are limited data on chemotherapy or treatment, and related toxicities, to dissect the impact of chemotherapy and ART-induced DDIs on survival among patients with HIV and breast cancer.

## Health System and Social Factors

In SSA, HIV is typically managed locally, whereas cancer is managed in oncology centers in cities. These differences may pose logistical challenges for the patients and make it difficult for clinicians to comanage the two conditions. Limited oncologic or surgical expertise, lack of access to standard treatment (eg, because of transport costs to oncology centers), and unavailability of some treatment modalities such as radiotherapy (two thirds of SSA lack radiotherapy services^37,38^) may lead to higher cost of care in patients with HIV and breast cancer.^10^ The lack of universal health care in most SSA countries means that patients with HIV and breast cancer bear the costs of treatment for both diseases out-of-pocket, leading to delayed or incomplete/no treatment. Indeed, studies have shown lower relative dose intensity of chemotherapy in patients with HIV and breast cancer,^39^ which may lead to suboptimal therapy and potentially treatment failure and drug resistance.^35^ In addition, there are many unanswered questions regarding the role of intersectional stigma and psychological distress in patients with HIV and breast cancer,^40^ which may lead to their reduced adherence/abandonment of cancer treatment.

In conclusion, the anticipated increasing numbers of patients with HIV and breast cancer call for more research on reasons underlying survival disparities by HIV status. More funding is needed for large clinical cohorts of breast cancer (with and without HIV), with standardized collection of granular HIV-cancer treatment data, to fully understand treatment interactions and their implications on survival. Patients with HIV and breast cancer should be included in clinical trials, especially in settings with high numbers of patients with HIV and breast cancer (eg, South Africa [22%],^8^ Namibia [15%], and Botswana [>20%]^39,41^), to enhance external generalizability of efficacy and safety of cancer-directed therapy in these patients. Addressing these gaps will hopefully translate to evidence-based interventions for improved outcomes in patients with HIV and breast cancer.
